# Account of a New Anæsthetic Agent, as a Substitute for Sulphuric Ether in Surgery and Midwifery

**Published:** 1848-01

**Authors:** J. Y. Simpson

**Affiliations:** Professor of Midwifery in the University of Edinburgh; Physician-Accoucheur to the Queen in Scotland, etc.


### ARTICLE III.

*Account of a New Anæsthetic Agent, as a Substitute for Sulphuric Ether in Surgery and Midwifery.* Commuuicated to the Medico-chirurgical Society of Edinburgh, at their Meeting on 10th November, 1847, by J. Y. Simpson, M.D., F. R.S.E., Professor of Midwifery in the University of Edinburgh; Physician-Accoucheur to the Queen in Scotland, etc.

From the time at which I first saw ether-inhalation successfully practised in January last, I have had the conviction impressed upon my mind, that we would ultimately find that other therapeutic agents were capable of being introduced with equal rapidity and success into the system, through the same extensive and powerful channel of pulmonary absorption. In some observations, which I wrote and published in February last, relative to the inhalation of sulphuric ether in midwifery, I stated that, in several obstetric cases, I had used ergot of rye in this way, along with ether.—(See *Monthly Journal of Medical Science*, p. 724.)

With various professional friends, more conversant with chemistry than I am, I have, since that time, taken opportunities of talking over the idea which I entertained of the probable existence or discovery of new therapeutic agents, capable of being introduced into the system by respiration, and the possibility of producing for inhalation vaporizable or volatile preparations of some of our more active and old established medicines: and I have had, during the summer and autumn, ethereal tinctures, &c., of several potent drugs, manufactured for me, for experiment, by Messrs. Duncan, Flockhart, & Co., the excellent chemists and druggists of this city.

Latterly, in order to avoid, if possible, some of the inconveniences and objections pertaining to sulphuric ether, (particularly its disagreeable and very persistent smell, its occasional tendency to irritation of the bronchi during its first inspirations, and the large quantity of it occasionally required to be used, more especially in protracted cases of labor,)—I have tried upon myself and others the inhalation of different other volatile fluids, with the hope that some one of them might be found to possess the advantages of ether, without its disadvantages. For this purpose, I selected for experiment and have inhaled several chemical liquids of a more fragrant or agreeable odor, such as the chloride of hydro-carbon (or Dutch liquid,) acetone, nitrate of oxide of ethyle (nitric ether,) benzin, the vapor of iodoform, &c.* I have found, however, one infinitely more efficacious than any of the others, viz. chloroform, or the perchloride of formyle, and I am enabled to speak most confidently of its superior anæsthetic properties, having now tried it upon upwards of thirty individuals. The liquid I have used has been

* In talking over, with different chemists, what fluids might be sufficiently volatile to be respirable, and hence deserving of being experimented upon, Mr. Waldie first named to me the perchloride of formyle as worthy, among others, of a trial; Dr. Gregory suggested a trial of the chloride of hydrocarbon, &c. I have been deeply indebted to Dr. Gregory and Dr. Anderson, for their kindness in furnishing me with the requisite chemical agents for these experiments; and also to my assistants, Dr. Keith and Dr. Duncan, for the great and hearty zeal with which they have constantly aided me in conducting the inquiry.

manufactured for me by Mr. Hunter, in the laboratory of Messrs. Duncan, Flockhart & Co.

Chloroform was first discovered and described at nearly the same time by Soubeiran (1831,) and Liebig, (1832;) its composition was first accurately ascertained by the distinguished French chemist, Dumas, in 1835. See the *Annales de Chimie et de Physique*, vols. xlviii, xlix, and lviii. It has been used by some practitioners internally; Guillot prescribed it as an antispasmodic in asthma, exhibiting it in small doses, and diluted 100 times. (See Bouchardat's *Annuaire de Therapeutique* for 1844, p. 35.) But no person, so far as I am aware, has used it by inhalation, or discovered its remarkable anæsthetic properties till the date of my own experiments.

It is a dense, limpid, colorless liquid, readily evaporating, and possessing an agreeable, fragrant, fruit-like odor, and a saccharine pleasant taste.

As an inhaled anæsthetic agent, it possesses over sulphuric ether the following advantages:

1. A greatly less quantity of chloroform than of ether is requisite to produce the anæsthetic effect; usually from a hundred to a hundred and twenty drops of chloroform only being sufficient; and with some patients much less. I have seen a strong person rendered completely insensible by six or seven inspirations of thirty drops of the liquid.

2. Its action is much more rapid and complete, and generally more persistent. I have almost always seen from ten to twenty full inspirations suffice. Hence the time of the surgeon is saved; and that preliminary stage of excitement, which pertains to all narcotizing agents, being curtailed, or indeed practically abolished, the patient has not the same degree of tendency to exhilaration and talking.*

* In practice I have found that any such tendency, even with ether, is avoided by, 1st, giving the patient from the first a large and overwhelming dose of the vapor, and 2ndly, by keeping him perfectly quiet and still, and preventing all noise and talking around him. I have elsewhere insisted on the importance of these points. (See the numbers of the Monthly *Journal of Medical Science* for March, 1847, p. 726, and for September, p.

3. Most of those who know from previous experience the sensations produced by ether inhalation, and who have subsequently breathed the chloroform, have strongly declared the inhalation and influence of chloroform to be far more agreeable and pleasant than those of ether.

4. I believe, that considering the small quantity requisite, as compared with ether, the use of chloroform will be less expensive than that of ether, more especially, as there is every prospect that the means of forming it may be simplified and cheapened.

5. Its perfume is not unpleasant, but the reverse; and the odor of it does not remain, for any length of time, obstinately attached to the clothes of the attendant, or exhaling in a disa-

154.) In the paper last referred to, I took occasion, when discussing the conditions requisite for insuring successful etherization, to observe, "*First,* The patient ought to be left, as far as possible, in a state of absolute quietude and freedom from mental excitement, both during the induction of etherization, and during his recovery from it. All talking and all questioning should be strictly prohibited. In this way any tendency to excitement is eschewed, and the proper effect of the ether inhalation more speedily and certainly induced. And, *Secondly,* with the same view, the primary stage of exhilaration should be entirely avoided, or at least reduced to the shortest possible limit, by impregnating the respired air as fully with the ether vapor as the patient can bear, and by allowing it to pass into the lungs both by the mouth and nostrils, so as rapidly and at once to superinduce its complete and anæsthetic effect; * * * * a very common but certainly a very unpardonable error being to exhibit an imperfect and exciting, instead of a perfect and narcotizing dose of the vapor. Many of the alleged failures and misadventures are doubtless entirely attributable to the neglect of this simple rule; not the principle of etherization, but the mode of putting it in practice being altogether to blame." But, *Thirdly,* whatever means or mode of etherization is adopted, the most important of the conditions required for procuring a satisfactory and successful result from its employment in surgery, consists in obstinately determining to avoid the commencement of the operation itself, and never venturing to apply the knife *until* the patient is under the influence of the ether-vapor, and *"thoroughly and indubitably soporized by it."* In fulfilling all these indications, the employment of chloroform evidently offers great and decided advantages, in facility and efficieney, over the employment of ether.

greeable form from the lungs of the patient, as so generally happens with sulphuric ether.

6. Being required in much less quantity, it is much more portable and transmissible than sulphuric ether.

7. No special kind of inhaler or instrument is necessary for its exhibition. A little of the liquid diffused upon the interior of a hollow-shaped sponge, or a pocket-handkerchief, or a piece of linen or paper, and held over the mouth and nostrils, so as to be fully inhaled, generally suffices in about a minute or two to produce the desired effect.*

I have not yet had an opportunity of using chloroform in any capital surgical operation, but have exhibited it with perfect success, in tooth-drawing,† opening abscesses, for annulling the

* When used for surgical purposes, perhaps it will be found to be most easily given upon a handkerchief, gathered up into a cup-like form in the hand of the exhibitor, and with the open end of the cup placed over the nose and mouth of the patient. For the first inspiration or two, it should be held at the distance of half an inch or so from the face, and then more and more closely applied to it. To insure a rapid and perfect anæsthetic effect—more especially where the operation is to be severe—one or two teaspoonsful of the chloroform should be at once placed upon the hollow of the handkerchief, and immediately held to the face of the patient. Generally a snoring sleep speedily supervenes; and when it does so, it is a perfect test of the superinduction of complete insensibility. But a patient may be quite anæsthetic without this system supervening.

† A young dentist who has himself had two teeth extracted lately—one under the influence of ether, and the other under the influence of chloroform—writes me the following statement of the results: "About six months ago I had an upper molar tooth extracted whilst under the influenco of ether, by Mr. Imlach. The inhalation was continued for several minutes before I presented the usual appearance of complete etherization; the tooth was then extracted; and, although I did not feel the leat pain, yet I was conscious of the operation being performed, and was quite aware when the crash took place. Some days ago I required another molar extracted on account of tooth-ache, and this operation was again performed by the same gentleman. I inhaled the vapor of chloroform, half a drachm being poured upon a handkerchief for that purpose, and held to my nose and mouth. Insensibility took place in a few seconds; but I was so completely *dead* this time, that I was not in the very slightest degree aware of any thing that took place. The subsequent stupifying effects of the chloroform went off more rapidly than those of the ether; and I was perfectly well and able again for my work in a few minutes."

pain of dysmenorrhœa and of neuralgia, and in two or three cases where I was using deep, and otherwise very painful galvano-puncture for the treatment of ovarian dropsy, &c. I have employed it also in obstetric practice with entire success. The lady to whom it was first exhibited during parturition, had been previously delivered in the country by perforation of the head of the infant, after a labor of three days' duration. In this, her second confinement, pains supervened a fortnight before the full time. Three hours and a-half after they commenced, and, ere the first stage of the labor was completed, I placed her under the influence of the chloroform, by moistening, with half a tea-spoonful of the liquid, a pocket handkerchief, rolled up into a funnel shape, and with the broad or open end of the funnel placed over her mouth and nostrils. In consequence of the evaporation of the fluid, it was once more renewed in about ten or twelve minutes. The child was expelled in about twenty-five minutes after the inhalation was begun. The mother subsequently remained longer soporose than commonly happens after ether. The squalling of the child did not, as usual, rouse her; and some minutes elapsed after the placenta was expelled, and after the child was removed by the nurse into another room, before the patient awoke. She then turned around and observed to me that she had "enjoyed a very comfortable sleep, and indeed required it, as she was so tired,* but would now be more able for the work before her." I evading entering into conversation with her, believing, as I have already stated, that the most complete possible quietude forms one of the principal secrets for the successful employment of either ether or chloroform. In a little time she again remarked that she was afraid her "sleep had stopped the pains." Shortly afterwards, her infant was brought in by the nurse from the adjoining room, and it was a matter of no small difficulty to convince the astonished mother that the labor was entirely

* In consequence of extreme anxiety at the unfortunate result of her previous confinement, she had slept little or none for one or two nights, preceding the commencement of her present accouchement.

over, and that the child presented to her was really her "own living baby."

Perhaps I may be excused from adding, that since publishing on the subject of ether inhalation in midwifery, seven or eight months ago,* and then for the first time directing the attention of the medical profession to its great use and importance in natural and morbid parturition, I have employed it, with few and rare exceptions, in every case of labor that I have attended; and with the most delightful results. And I have no doubt whatever, that some years hence the practice will be general. Obstetricians may oppose it, but I believe our patients themselves will force the use of it upon the profession.† I have never had the pleasure of watching over a series of better and more rapid recoveries; nor once witnessed any disagreeable result follow to either mother or child; whilst I have now seen an immense amount of maternal pain and agony saved by its employment. And I most conscientiously believe that the proud mission of the physician is distinctly twofold—namely, to alleviate human suffering, as well as preserve human life.

### *Chemical Constitution of Chloroform.*

Formyle is the hypothetical radical of formic acid. In the red ant (*Formica rufa*) formic acid was first discovered, and hence its name. Gehlen pointed it out as a peculiar acid; and it was afterwards first artificially prepared by Doebereiner. Chemists have now devised a variety of processes, by which formic acid may be obtained from starch, sugar, and, indeed, most other vegetable substances.

A series of chlorides of formyle are produced when chlorine and the hypochlorites are brought to act on the chloride, oxide,

* See *Monthly Journal of Medical Science* for February, p. 639; for March, p. 718 and 721, and April, p. 794, &c.

† I am told that the London physicians, with two or three exceptions only, have never yet employed ether inhalation in their midwifery practice. Three weeks ago, I was informed in a letter from Professor Montgomery of Dublin, that he believed that in that city, up to that date, it had not been used in a single case of labor.

and hydrated oxide of methyle, (pyroxylic or wood spirit.) In the same way as formic acid may be artificially procured from substances which do not contain formyle ready formed, so also are the chlorides of this radical capable of being procured from substances which do not originally contain it.

Chloroform, chloroformyle, or the perchloride of formyle, may be made and obtained artificially by various processes—as by making milk of lime, or an aqueous solution of caustic alkali act upon chloral—by distilling alcohol, pyroxylic spirit, or acetone, with chloride of lime—by leading a stream of clorine gas into a solution of caustic potass in spirit of wine, &c. The preparation which I have employed, was made according to the formula of Dumas:—

| | | |
|---|---|---|
| "℞ Chloride of lime in powder, . | ℔ | iv. |
| Water, . . . . | ℔ | xii. |
| Rectified spirit, . . . | f ℥ | xii. |

"Mix in a capacious retort or still, and distil as long as a dense liquid, which sinks in the water with which it comes over, is produced."—(Gray's *Supplement to the Pharmacopœia*, 1846, p. 633.)

The resulting perchloride of formyle consists of two atoms of carbon, one of hydrogen, and three of chlorine. Its specific gravity is much greater than that of water, being as high as 1.480. It boils at 141°. The density of its vapor is 4.2. It is not inflammable; nor changed by distillation with potassium, potash, sulphuric, or other acids. (See Turner's *Elements of Chemistry*, 8th edition, p. 1009; Gregory's *Outlines of Chemistry*, part ii. p. 401; Fownes' *Manual of Elementary Chemistry*, p. 419; Thomson's *Chemistry of Organic Bodies*, p. 312; Loewig's *Organische Chemie*, vol. i. p. 498.)

It is now well ascertained that three compound chemical bodies possess, when inhaled into the lungs, the power of superinducing a state of anæsthesia, or insensibility to pain in surgical operations, &c., namely, nitrous oxide, sulphuric ether, and perchloride of formyle. The following tabular view shows that these agents are entirely different from each other in their chemical constitution, and hence that their elementary composition affords no apparent clue to the explanation of their anæsthetic properties :—

| | Propor. of Nitrogen. | Propor. of Oxygen. | Propor. of Carbon. | Propor. of Hydrogen. | Propor. of Chlorine. |
|---|---|---|---|---|---|
| Nitrous Oxide, | 1 Atom. | 1 Atom. | ... | ... | ... |
| Sulphuric Ether, | ... | 1 Atom. | 4 Atoms. | 5 Atoms. | ... |
| Chloroform, | ... | .. | 2 Atoms. | 1 Atom. | 3 Atoms. |

It is perhaps not unworthy of remark, that when Soubeiran, Liebig, and Dumas engaged, a few years back, in those inquiries and experiments by which the formation and composition of chloroform was first discovered, their sole and only object was the investigation of a point in philosophical chemistry. They labored for the pure love and extension of knowledge. They had no idea that the substance to which they called the attention of their chemical brethren could or would be turned to to any *practical* purpose, or that it possessed any physiological or therapeutic effects upon the animal economy. I mention this to show, that the *cui bono* argument against philosophical investigations on the ground that there may be at first no apparent practical benefit to be derived from them, has been amply refuted in this, as it has been in many other instances. For I feel assured, that the use of chloroform will soon entirely supersede the use of ether; and, from the facility and rapidity of its exhibition, it will be employed as an anæsthetic agent in many cases, and under many circumstances, in which ether would never have been had recourse to. Here then we have a substance which, in the first instance, was merely interesting as a matter of scientific curiosity and research, becoming rapidly an object of intense importance, as an agent by which human suffering and agony may be annulled and abolished, under some of the most trying circumstances in which human nature is ever placed.

P. S. Since the above observations were sent to the press, I have—through the great kindness of professor Miller and Dr. Duncan—had an opportunity of trying the effects of the inhalation of chloroform, to-day, in three cases of operation in the Royal Infirmary of Edinburgh. A great collection of professional gentlemen and students witnessed the results, and among the number was professor Dumas of Paris, the chemist who first ascertained and established the chemical composition of chloroform. He happened to be passing through Edinburgh, engaged along with Dr. Milne Edwards, who accompanied him, in an official investigation for the French government—and was, in no small degree, rejoiced to witness the wonderful physiological effects of a substance with whose chemical history his own name was so intimately connected.

I append notes, obligingly furnished to me by professor Miller and Dr. Duncan, of the three cases of operation. The two first cases were operated on by professor Miller; the third by Dr. Duncan. In applying the chloroform in the first case, I used a pocket handkerchief as the inhaling instrument; in the two last I employed a hollow sponge.

Case 1.—"A boy, four or five years old, with necrosis of one of the bones of the fore-arm. Could speak nothing but Gaelic. No means, consequently, of explaining to him what he was required to do. On holding a handkerchief, on which some chloroform had been sprinkled, to his face, he became frightened, and wrestled to be away. He was held gently, however, by Dr. Simpson, and obliged to inhale. After a few inspirations he ceased to cry or move, and fell into a sound snoring sleep. A deep incision was now made down to the diseased bone; and, by the use of the forceps, nearly the whole of the radius, in the state of sequestrum, was extracted. During this operation, and the subsequent examination of the wound by the finger, not the slightest evidence of the suffering of pain was given. He still slept on soundly, and was carried back to his ward in that state. Half an hour afterwards, he was found in bed, like a child newly awakened from a refreshing sleep, with a clear merry eye, and placid expression of countenance, wholly unlike what is found to obtain after ordinary etherization. On being questioned by a Gaelic interpreter who was found among the students, he stated that he had never felt any pain, and that he felt none now. On being shown his wounded arm, he looked much surprised, but neither cried nor otherwise expressed the slightest alarm."

Case 2.—"A soldier who had an opening in the cheek—the result of exfoliation of the jaw—was next made to inhale. At first he showed some signs of moving his hands too freely; but soon also fell into a state of sleep and snoring. A free incision was made across the lower jaw, and from this the dense adhering integuments were freely dissected all round, so as to raise the soft parts of the cheek. The edges of the opening were then made raw, and the whole line of incision was brought together by several points of suture. This patient had previously undergone two minor operations of a somewhat similar kind; both of them had proved unsuccessful, and he bore them very ill—proving unusually unsteady, and complaining bitterly of severe pain. On the present occasion, he did not wince or moan in the slightest degree; and, on the return of consciousness, said that he had felt nothing. His first act, when apparently about half awake, was suddenly to clutch up the sponge with which the chloroform was used, and readjust it to his mouth, obviously implying that he had found the inhalation from it any thing but a disagreeable duty.

"This case was further interesting as being one of those operations in the region of the mouth, in which it has been deemed impossible to use ether—and, certainly, it would have been impossible to have performed the operation with any complicated inhaling apparatus applied to the mouth of the patient."

Case 3.—"A young man of about twenty-two years of age, having necrosis of the first phalanx of the great toe, and ulceration of the integuments, the consequence of injury. The ulcerated surface was exceedingly tender to the touch—so much so, that he winced whenever the finger was brought near to it; and the slightest pressure made him cry out. After the removal of the dressings, which caused some pain and fretting, the inhalation was commenced, and the patient almost immediately* became insensible, and lay perfectly still, while the diseased mass was being removed by amputation of the toe through the middle of the second phalanx. The inhalation was now stopped. The edges of the wound were then brought together with three stitches, and the wound dressed. The patient shortly afterwards awoke, looked round him, and gratefully declared his entire and perfect freedom from all pain and uneasiness during the operation."

The whole quantity of chloroform used in these three operations did not exceed half an ounce—and, as professor Miller afterwards observed to the students that were present, if ether had been used, several ounces of it would have been requisite to produce the same amount of anæsthetic effect.

The following case occurred also to-day, to Dr. Miller, in private practice. The notes of it and the subsequent remark are in his own words.

Case 4.—"A young lady wished to have a tumor (encysted) dissected out from beneath the angle of the jaw. The chloroform was used in small quantity (about a drachm,) sprinkled upon a piece of operation sponge. In considerably less than a minute she was sound asleep, sitting easily in a chair, with her eyes shut, and with her ordinary expression of countenance. The tumor was extirpated, and a stitch inserted, without any pain having been either shown or felt. Her sensations, throughout, as she subsequently stated, had been of the most pleasing nature; and her manageableness during the operation was as perfect as if she had been a wax doll or a lay figure.

"No sickness, vomiting, headache, salivation, uneasiness of chest, in any of the cases. Once or twice a tickling cough took place in the first breathings."

I have, up to this date, exhibited the chloroform to about fifty individuals. In not a single instance has the slightest bad result of any kind whatever occurred from its employment.

Edinburgh, *November* 15, 1847.

* Dr. Christison, who was watching the result, informs me that this patient was affected in half a minute.

